# Lectures on Auto-Intoxication in Disease, or Self-Poisoning of the Individual

**Published:** 1894-09

**Authors:** 


### Book Reviews.

LECTURES ON AUTO-INTOXICATION IN DISEASE, OR SELF-POISONING OF THE INDIVIDUAL. By Chas. Bouchard, Professer of Pathology and Therapeutics, Member of the Academy of Medicine, and Physician to the Hospitals Paris. Translated with a preface by Thomas Oliver, M.A., M.D., F.R.C.P., Professor of Physiology, University of Durham; Physician to the Royal Infirmary, Newcastle-upon-Tyne; and Examiner in Physiology, Conjoint Board of England, Philadelphia. F. A. Davis Co., London: F. J. Rebman, 1894.

Until quite recently there has appeared but little literature of value on this important subject of "Auto-intoxication in Disease or the Self-poisoning of the Individual." Professor Bouchard's book is timely and valuable. "No subject commands a greater interest, none demands a more serious study." That the poisons generated within the body are potent factors for evil in health, and especially in certain diseased conditions, is a well known fact, but the nature of the poisons generated, and their effect upon the economy when allowed to accumulate, are facts which, until now, have been little understood.

The pathologists and bacteriologists have been slow in giving their support to the theories and opinions of this great and original worker. But at last he is being properly accorded the consideration which he so surely deserves, and this his latest book has taken rank as the greatest of its kind, and is universally spoken of in terms of highest praise

To Selmi, Vaughn, Pasteur, and others, Bouchard has left the classification, nomenclature, and histories of the micro-organisms, giving but little space to these subjects, but goes at once into the general action of these animal poisons, and the conditions of the economy which nourish or check their development. The chapters on "Pathogenic Processes," "Toxicity of Urines," "Auto-intoxication of Intestinal Origin," "Typhoid Fever," and others are teeming with practical suggestions, and every page of this valuable little book is filled with facts incontestable and invaluable, which we can no longer afford to ignore or refuse to understand.

"What is characteristic of the present day, so far as medicine is concerned, is the high place we assign to the origin of disease." To all who seek to keep up with the wonderful discoveries and add to their knowledge of pathological histology, of symptomatology and of therapeutics, the origin of disease and the processes which continue it, these lectures of Professor Bouchard are indispensable.

